# Short-Term Impact of Slow Maxillary Expansion on Labial Ectopic Canine Eruption Pathway in Children: A Retrospective Study

**DOI:** 10.3390/children12050653

**Published:** 2025-05-19

**Authors:** Qian Tong, Xue Yang, Yue Fei, Jun Wang

**Affiliations:** 1 Department of Pediatric Dentistry, Shanghai Ninth People’s Hospital, Shanghai Jiao Tong University School of Medicine, Shanghai 200011, China; tongqian0915@163.com (Q.T.); 72300116141@shsmu.edu.cn (X.Y.); 124031@sh9hospital.org.cn (Y.F.); 2 College of Stomatology, Shanghai Jiao Tong University, Shanghai 200125, China; 3 National Center for Stomatology, Shanghai 200125, China; 4 National Clinical Research Center for Oral Diseases, Shanghai 200125, China; 5 Shanghai Key Laboratory of Stomatology, Shanghai 200125, China

**Keywords:** SME, labial ectopic canine, eruption pathway

## Abstract

**Objectives:** This retrospective study evaluated the short-term effects of removable slow maxillary expansion (SME) on eruption patterns of labially ectopic canines in a Chinese pediatric population, comparing treated patients with untreated controls. **Methods:** Seventy-six patients (mean age 8.38 ± 0.88 years) underwent SME treatment for 11.04 ± 4.44 months. Canine positions were categorized as labial ectopic (TE: *n* = 40) or normally positioned (TN: *n =* 112). The TE group was stratified vertically: superior (TES; *n =* 15, canines above lateral incisors’ roots or adjacent to unerupted incisors) and inferior (TEI; *n =* 25, canines adjacent to erupted lateral incisors’ roots). Untreated controls (*n =* 58; mean age 8.46 ± 0.78 years) included labial ectopic (CE group; *n =* 32) and normal canines (CN group; *n =* 84), with CE further divided vertically into CES (*n =* 24) and CEI (*n =* 8). Panoramic radiographs at baseline (T0) and follow-up (T1) evaluated sector distribution, midline proximity (3c-ML: canine cusp to midline distance), vertical position (3c-OP: cusp to occlusal plane distance), and angular (3^ML: canine-midline angle). **Results:** SME significantly improved midline proximity (3c-ML increased) while reducing vertical height (3c-OP decreased) and angulation (3^ML reduced) in the TE group. Notably, TE patients revealed a significantly greater increase in 3c-ML compared to CE. Subgroup analysis showed that TEI canines exhibited significant improvements in all three parameters (3c-OP, 3c-ML, and 3^ML), whereas TES canines displayed minimal changes. The shifts in sector distribution were similar between the treatment and control groups. **Conclusions:** SME demonstrated short-term efficacy in guiding labially ectopic canines toward more favorable eruption trajectories, particularly when erupted beyond the roots of the lateral incisor. The observed positional improvements underscore SME’s potential to optimize eruption outcomes during early orthodontic intervention.

## 1. Introduction

Ectopic eruption of permanent maxillary canines is a common developmental anomaly in dentition, characterized by the deviation of canines from their normal eruption path, resulting in their emergence either in the palatal or labial direction [1,2]. In Asian populations, labial ectopic eruptions are more prevalent than palatal ones, with reported ratios ranging from 2:1 to 3:1 [3,4]. Conversely, among Caucasian populations, palatal impaction is more common than buccal impaction, with ratios ranging from 3:1 to 6.6:1 [5,6]. The etiology of palatal impactions is often explained by the guidance theory and genetic predisposition, whereas buccal impactions are typically attributed to space deficiencies [7]. In Chinese patients, anterior transverse deficiency has been identified as a significant contributing factor to buccal canine impaction [2].

It is generally accepted that labially displaced canines have a higher likelihood of spontaneous eruption [8,9]. However, studies have demonstrated that during the ectopic eruption of canines, root resorption of adjacent lateral incisors occurs in 27% to 49.5% of cases. Although the precise mechanism of this root resorption is unclear, it is widely believed to be associated with the physical proximity of the ectopic canine and the increased force exerted on the lateral incisor root [3,10,11]. Several potential risk factors have been proposed, including patient gender, the position of the canine apex, vertical position of the canine crown, canine magnification, and the distance of the canine from the midline [12,13,14].

Early intervention strategies for ectopic canines include interceptive procedures such as extraction of deciduous canines or orthodontic treatments aimed at maintaining or increasing the maxillary arch length or perimeter, with examples including cervical headgear and arch expansion techniques [15,16,17,18]. Despite this, research specifically addressing interceptive treatments for labial ectopic canines remains limited. Harada-Karashima et al. found that rapid maxillary expansion (RME) in the early mixed dentition was effective in managing labially impacted maxillary canines [19]. However, RME has several clinical limitations, including potential posterior tooth tipping, increased risk of root resorption, and significant alterations in nasal width and midfacial soft tissues that may lead to aesthetic concerns [20,21,22]. In contrast, slow maxillary expansion (SME) has been widely used in early treatment [23,24,25,26], with its biomechanical characteristics providing clinical advantages. SME applies gentle continuous forces (0.25–0.5 N), reducing posterior tooth tipping risk by 32% compared to RME [22,27], while decreasing root resorption incidence by 62% [20], thereby minimizing periodontal damage. Additionally, SME induces more subtle changes in nasal width and midfacial soft tissues [21], effectively avoiding aesthetic issues like alar base widening associated with rapid expansion, making it particularly suitable for young children and aesthetic-sensitive patients. Regarding long-term stability, SME demonstrates less post-retention arch width loss [28], with its biocompatibility advantages establishing it as the preferred option for early intervention in mixed dentition. Despite its known advantages, it remains unclear whether SME can effectively improve the intraosseous position of labially impacted maxillary canines in the short term, particularly with regard to angular changes, vertical displacement, and proximity to the midline, reducing the risk of root resorption during ectopic eruption, and thereby minimizing the complexity and complications. This knowledge gap limits clinicians’ comprehensive understanding and application of SME for labial ectopic canines. To date, no studies have addressed this issue.

Furthermore, the three-dimensional (3D) eruption trajectory and dynamic angular changes in maxillary canines are influenced not only by alveolar bone morphology and available space but also by the anatomical positioning of adjacent teeth (e.g., lateral incisors) [29,30]. The biomechanical impact of SME may differ depending on the developmental stage of labial ectopic canines—whether the canine crown has not yet reached the lateral incisor root apex level or has already shifted to the lateral aspect of the incisor root.

Therefore, this study aims to evaluate the influence of SME on the eruption path of labially ectopic maxillary canines in mixed dentition through panoramic radiograph analysis, addressing current research deficiencies. In addition, this investigation will innovatively explore whether SME produces differential effects on maxillary canines at various eruption stages. These findings may offer clinicians more precise guidance on intervention timing and treatment strategies to optimize therapeutic outcomes for labial ectopic canines.

## 2. Materials and Methods

### 2.1. Study Design

This study is a retrospective cohort study. The study protocol was approved by the Ethics Committee of Shanghai Ninth People’s Hospital, affiliated with Shanghai Jiao Tong University School of Medicine (Approval No. SH9H-2024-T189-1). Eligible subjects were retrospectively selected from the records of patients enrolled in the Growth and Development Management Program between September 2019 and December 2023. The study compared outcomes between a treatment group receiving removable SME and an untreated control group, with longitudinal evaluation of canine positional changes.

### 2.2. Samples

Sample size was calculated using PASS software (version 15.0.3) considering the changes in 3^ML as the primary outcome on the data of a preliminary selected pilot sample of 10 patients (5 patients per group). To retrieve β = 0.20 with α set at 0.05, a sample of at least 63 subjects per group was necessary. The final study cohort exceeded this requirement, comprising 152 treated sides (76 patients) and 116 control sides (58 patients).

The treatment group included 76 patients who received removable SME therapy in the Department of Pediatric Dentistry. The inclusion criteria were as follows: (1) Chinese population; (2) patients in the mixed dentition stage; (3) presence of unerupted maxillary canines; (4) good compliance, with more than 5 mm of expansion achieved using a removable expander; and (5) canine position confirmed by anteroposterior radiographs, CBCT scans, or intraoral photographs obtained during clinical follow-up. Exclusion criteria were as follows: (1) a history of orthodontic treatment; (2) presence of supernumerary teeth or lateral incisor hypoplasia; (3) early loss of deciduous canines; (4) oral diseases associated with dental trauma or eruption disturbances; (5) craniofacial anomalies or syndromes; and (6) incomplete or poor-quality imaging data.

The control group comprised 58 patients who did not undergo orthodontic treatment, and their canine position was confirmed by anteroposterior radiographs, CBCT scans, or intraoral photographs during follow-up. Each patient had at least two panoramic X-rays taken six months apart. The exclusion criteria for the control group were identical to those for the treatment group.

### 2.3. Treatment Protocol and Imaging

The treatment protocol for the treated group involved the activation of a screw by a quarter turn (0.25 mm) two to three times a week until the palatal cusps of the maxillary posterior teeth achieved contact with the buccal cusps of the mandibular posterior teeth. Upon achieving the desired expansion, the expansion screw was secured with glass ionomer cement. Subsequently, the original appliance was worn for 3 months as a retention phase. Panoramic radiographs (OPGs) were taken before treatment (T0) and at the end of the retainment (T1).

OPGs were captured using an Orthophos XG 3 device (Dentsply Sirona, Bensheim, Germany). Panoramic X-rays were acquired by experienced radiologists in the radiology department of our hospital. A bite locator was employed to ensure centric occlusion of the patient’s upper and lower teeth, and the patient’s head was positioned to maintain the Frankfort horizontal plane parallel to the ground.

### 2.4. Data Acquisition and Measurement

**Figure 1:**
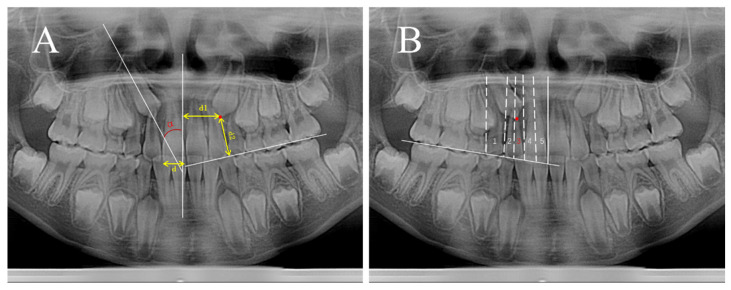
(**A**): Angular and distance measurements of canines. The α angle represents the angle between the long axis of the canine and the midline. Measurements include the perpendicular distance from the tip of the canine to the midline (d1) and to the occlusal plane (d2). The width of the maxillary central incisor (d) was used as a reference measurement, standardized to 10 units. Distances (d1, d2) were adjusted proportionally relative to the width of the central incisor (d). (**B**): Distribution of canine sector based on the location of the canine tip. The numbers 1–5 represent the sector, and the red dots indicate the canine cusp. The canine in this figure is situated in sector 3.

Digital panoramic images taken at T0 and T1 were analyzed by an experienced orthodontist. The following measurements improved from Ericson and Kurol [15,31] were made on the panoramic radiographs: (1) distance: canine cusp-to-occlusal plane distance (3c-OP) and canine cusp-to-midline distance (3c-ML); (2) angle: canine-to-midline angle (3^ML) (Figure 1A); (3) sector: representing the position of the canine cusp relative to the central and lateral incisors. The assessment encompassed 5 sectors, where sector 1 denoted the canine cusp located posteriorly to the distal aspect of the lateral incisor, while sector 5 corresponded to the mesial half of the upper central incisor (Figure 1B).

The occlusal plane was identified as a horizontal line tangentially connecting the mesio-buccal cusp tip of the first permanent molar with the incisal margin of the maxillary first permanent incisor. The midline was defined as the perpendicular line passing through the point of the anterior nasal ridge. To obviate the need for linear measurements on the OPG, the width of the maxillary central incisor was established as a reference measurement, standardized to 10 units. Subsequently, the distance was adjusted proportionally to the width of the central incisor [32].

Canine positions were evaluated using Dolphin Imaging (version 11.95) through a customized radiographic analysis. All measurements were conducted by a single blinded observer to avoid systematic bias from inter-observer differences or unmasked expectations. To minimize intra-observer variability, 50 orthopantomograms (OPGs) were measured with a two-week interval for calibration prior to conducting the study measurements. A second measurement was performed two months after the initial assessment to confirm the examiner’s reliability over time and to reduce concerns about drift in measurement criteria.

### 2.5. Classification

Canines in the treatment group and control group were categorized based on specific criteria to delineate ectopic canines. Ectopic canines were identified as those with the canine cusp located in sectors 2, 3, 4, or 5, while canines positioned in sector 1 were considered normally positioned [33]. In the treatment group, ectopic canines were designated as TE (ectopic canine in treatment group) and normally positioned canines as TN (normally positioned canine in treatment group). Similarly, in the control group, ectopic canines were termed CE (ectopic canine in control group), and normally positioned canines as CN (normally positioned canine in control group). Additionally, considering the dynamic changes in canine eruption direction during different eruption stages [29], canines were further classified based on the relative position of the canine cusp tip and the root of the lateral incisor. Canines located above the root apex of the lateral incisors or adjacent to non-erupted lateral incisors were classified as superior positioned, whereas those adjacent to the roots of erupted lateral incisors were classified as inferior positioned. In the TE group, superior-positioned canines before treatment were referred to as TES (superior-positioned canines in the TE group) and inferior-positioned canines before treatment as TEI (inferior-positioned canines in the TE group). In the CE group, superior-positioned canines before treatment were termed CES (superior-positioned canines in the CE group) and inferior-positioned canines before treatment as CEI (inferior-positioned canines in the CE group).

After grouping, the evaluation parameters within each group were compared before and after treatment. Additionally, the changes (T1 − T0) of measurements were compared between the treatment and control groups to discern any discrepancies.

### 2.6. Statistical Analysis

To evaluate the measurement method’s error, Cohen’s kappa and Intraclass Correlation Coefficients (ICC) were calculated. Baseline characteristics between the two groups were compared using the chi-squared test for categorical variables and the independent samples *t*-test for continuous variables. Pearson’s chi-square test was applied to compare the sectors of canines before and after treatment, while Logistic regression analysis was employed to compare the sector changes between groups. Intragroup comparisons of distance and angular measurements between T0 and T1 were performed using a combination of Paired Sample *t*-test and Wilcoxon signed-rank test. Intergroup comparisons between the treatment and control groups were conducted using the Mann–Whitney U test and Independent Samples *t*-test. Statistical analysis was performed using SPSS software (IBM SPSS Statistics 21). Statistical significance was set as *p* < 0.05.

## 3. Results

Cohen’s kappa (0.902) and the intraclass correlation coefficient (0.955–0.991) demonstrated reliable ranking and measurement procedures.

Seventy-six individuals were included in the treatment group, while the control group consisted of 58 individuals. The distribution of gender, age, and treatment/observation period among the two groups is illustrated in Table 1, revealing no statistically significant differences between them. Due to asymmetry, statistical analyses of measurement results were conducted at the tooth level. Table 2 presents the distribution of sectors in the two groups before and after treatment. Following a period of treatment/observation, intra-group comparisons demonstrated improvements in sector distribution for both groups. However, inter-group comparisons did not yield statistically significant differences, suggesting comparable changes in sector distribution between the treatment and control groups.

Table 3 provides a summary of the canine position at T0 and T1, as well as the mean change (T1 − T0). Both the treatment and control groups showed statistically significant changes in 3c-OP, 3c-ML, and 3^ML. Notably, in the T1 − T0 category, the TE group displayed larger changes compared to the CE group in 3c-ML and 3^ML, suggesting that the TE group showed a greater increase in movement away from the midline and distal up-righting. On the other hand, the CE group exhibited higher variability in 3c-OP. In the TES group, none of the changes were significant, while all changes in the TEI were found to be statistically significant.

Table 4 presents the intergroup comparisons of the change values (T1 − T0) in measurement indicators. Comparisons between the TE and CE groups showed statistically significant differences in 3c-ML (*p* = 0.043). No significant statistical difference was observed between the TES and CES groups. Comparing the TES and TEI groups, significant differences were found in the change values of 3c-ML and 3^ML.

## 4. Discussion

The eruption pathway of maxillary canines follows a complex and convoluted trajectory [29,34]. Given the potential complications, such as root resorption of adjacent teeth caused by abnormal intraosseous eruption pathways, it is essential to explore whether interceptive treatments can facilitate the timely adjustment of ectopic canines’ intraosseous position and eruption pathway [3,33]. While much attention has been directed toward interventions like RME to enhance the eruption rate of palatally impacted canines [18,35,36], few studies have focused on evaluating changes in intraosseous positioning or the correction of abnormal eruption pathways. Some studies have explored the effects of SME on the eruption pathway of ectopic canines [31,37]. Caprioglio et al. retrospectively compared the effects of SME using a Quad-helix with RME using the Haas expander and found that SME did not improve the angle of palatally impacted canines relative to the midline [37]. Conversely, Willems et al. reported that early SME improved the canine’s angle to the midline and its position within specific sectors, although their study did not differentiate between labial and palatal ectopic canines [31]. This distinction is clinically significant, given the distinct etiological and positional characteristics of labial and palatal ectopic canines [7,38], highlighting the need to specifically evaluate the effects of expansion treatment on labially displaced canines. One study has examined the effects of RME on labial ectopic canines [19]. Additionally, the rate of expansion, short-term skeletal expansion effects, and alveolar bone remodeling differ between SME and RME [25,39,40], which may influence treatment outcomes. These gaps underscore the importance of investigating SME’s effect on labial ectopic canines, particularly across different eruption stages.

In this study, we assessed canine eruption pathways using four indicators: sector, distance from the occlusal plane (3c-OP), distance from the midline (3c-ML), and the angle between the canine and the midline (3^ML).

A notable finding was the significant increase in the 3c-ML distance in the treatment group (TE), with a mean displacement of 2.06, compared to a slight and non-significant increase of 0.50 in the control group (CE) (Table 3). The between-group comparison revealed a statistically significant difference (*p* = 0.043) (Table 4), suggesting that SME facilitates lateral displacement of labially ectopic canines away from the midline.

Clinically, this lateral movement is critical. Previous studies have identified a canine-lateral incisor proximity of less than 1 mm as a risk factor for root resorption [11]. The 2.06 displacement observed in the TE group may therefore represent a clinically meaningful threshold for establishing inter-radicular clearance, thereby reducing the risk of root resorption and dental overlap. Subgroup analysis further revealed that this effect was location-dependent. The TEI subgroup demonstrated a significant increase in the 3c-ML distance (3.06), while the TES subgroup exhibited minimal, non-significant change (0.38) (Table 3). These findings suggest that SME is more effective at promoting lateral movement when the canine has already descended to the level of or beyond the lateral incisor root apex. Our results are in contrast to those reported by Willems et al. [31], who reported no significant increase in the 3c-ML distance following slow expansion, with a marked increase instead observed in their control group. This discrepancy may be explained by differences in study design, particularly regarding the inclusion criteria. Unlike Willems et al., who did not differentiate between labially and palatally displaced canines, our study specifically targeted labially ectopic canines, which may respond differently to SME.

Further analysis of angular changes showed that in the TE group, the 3^ML angle significantly decreased (4.21°) following SME treatment, while only a slight, non-significant increase (1.4°) was observed in the CE group (Table 3). This suggests that SME treatment facilitates the uprighting of labial ectopically positioned canines, resulting in a more vertical alignment. A reduced 3^ML angle is clinically meaningful, as it indicates a more vertically oriented canine, which helps separate it from neighboring anatomical structures, reducing the risk of dilaceration and eruption complications [41]. These results align with those of Karashima et al. [19], who found that RME reduced the 3^ML angle of labially ectopic canines across various age groups (7–10 years). Conversely, Caprioglio et al. reported an increase in the 3^ML angle in both the SME group (treated with the Quad-helix appliance) and the observation group for palatally displaced canines over a 12-month period [37]. These contrasting results suggest that the positional adjustments of labially ectopic canines differ from those of palatally ectopic canines following expansion treatment. This divergence may arise from the varying resistance encountered during tooth movement, which is likely influenced by the canine’s initial position within the bone. Stratified analysis by eruption stage revealed that the 3^ML angle decreased significantly in the TEI and CEI subgroups (by 6.41° and 4.24°, respectively), while only minimal reductions were observed in the TES and CES groups (0.53° and 0.45°, respectively) (Table 3). A statistically significant difference in the 3^ML angle (*p* = 0.000) was observed between the TES and TEI groups (Table 4). These results indicate that SME is more effective in altering canine inclination when the tooth is positioned inferiorly.

The differential outcomes of 3c-ML and 3^ML between the TEI and TES groups may be multifactorial. During physiological eruption, superior-positioned canines tend to tilt further toward the midline, while canines that continue erupting tend to become more distally upright [29]. In this context, the impact of SME may be insufficient to overcome or reverse the inherent mesial inclination of teeth. The observed improvements in ectopic canine eruption pathways following SME may be attributed to increased alveolar space created post-expansion, facilitating canine eruption adjustments. However, the influence of maxillary expansion on alveolar bone width is less pronounced in superior regions compared to inferior areas [42]. As a result, canines in superior positions benefit less from SME in terms of spatial accommodation, limiting the potential for angular or positional correction. Anatomical constraints further contribute to this limitation. Canines located near the maxillary sinus or nasal floor often face increased cortical bone resistance. In such cases, the modest alveolar remodeling achieved through SME may provide insufficient space to allow meaningful positional changes. Additionally, the eruptive forces of canines at early stages of eruption tend to be weaker, lacking the physiological momentum necessary to facilitate synergistic repositioning even when expansion is applied.

While existing studies propose varying chronological benchmarks for optimal RME efficacy, our findings emphasize the significance of dental developmental stages over chronological age. Harada-Karashima et al. [19] identified the 7–8-year age range as optimal for treating labially impacted canines, aligning with Mutinelli et al.’s [43] recommendation for initiating RME before permanent lateral incisor eruption (typically around age 8). Similarly, Baccetti et al.’s [44] comparative analysis demonstrated superior treatment outcomes in early mixed dentition versus late mixed dentition phases. However, our clinical observations reveal substantial individual variability in canine eruption patterns among patients of the same chronological age. Notably, vertical eruption stages of canines showed stronger correlation with treatment outcomes than numerical age parameters. This suggests that the developmental status of the canine may be a more reliable indicator for determining the optimal timing of SME than conventional age-based benchmarks.

Across all groups, a reduction in the 3c-OP distance was observed (Table 3). Notably, the reduction in the TE group was smaller than in the CE group, consistent with previous studies [31]. This result indicates that in ectopically erupted canines, maxillary expansion treatment may lead to more focused distal uprighting and correction of the eruption direction, rather than movement toward the occlusal plane in the short term.

Regarding sector changes, our study found limited improvement in sector distribution following SME treatment compared to the control group. Although significant improvement occurred from sector 2 to sector 1, no substantial improvements were observed in canines located in sectors 3 or 4; in some cases, the condition worsened. This aligns with Sigler et al., who found that ectopic canines treated successfully with rapid arch expansion were primarily located in sectors 1 and 2 [36].

This study retrospectively investigated the effects of early SME treatment on labially displaced canines in patients. The results demonstrated that SME significantly facilitated distal uprighting and lateral displacement of canines away from the midline. Notably, canines erupting lateral to the lateral incisors showed more pronounced improvement. These results suggest that early detection of canine ectopia through clinical examination and even radiological imaging, followed by early intervention, can effectively improve the eruption pathway of labially ectopic canines within the bone and reduce the likelihood of related complications. Furthermore, the findings highlight the clinical significance of treatment timing, suggesting that intervention with SME should be implemented when labially ectopic canines reach the lateral aspect of the lateral incisors during eruption. The analysis demonstrated that canine positional improvement predominantly occurred in sector 2 cases, while those located in sectors 3 or 4 showed limited therapeutic response, with select cases progressing to more severe displacement. It implies that expansion alone may be insufficient for managing severely ectopic canines; therefore, combined therapies such as expansion with deciduous canine extraction or surgical exposure with orthodontic traction should be considered.

Notwithstanding these findings, several methodological limitations inherent to retrospective research should be acknowledged. First, the non-randomized, observational nature of the study limits causal inference and introduces potential selection bias. Second, the absence of standardized metrics for maxillary arch width and crowding severity across study groups may have confounded the assessment of eruption patterns. Third, subgroup analyses were constrained by limited sample sizes, reducing statistical power and increasing the risk of type II errors. Lastly, reliance on panoramic radiographs—while common in orthodontic settings—introduces technical limitations, including distortion, anatomical superimposition, and magnification errors [31], which may affect the precision of three-dimensional positional assessments. These limitations highlight the need for prospective, longitudinal studies employing standardized measurements and three-dimensional imaging modalities to validate and extend the current findings. Such research will be instrumental in refining treatment protocols and optimizing outcomes for patients with labially ectopic maxillary canines.

## 5. Conclusions

SME demonstrates short-term efficacy in improving the eruption trajectory of labially ectopic maxillary canines. By enhancing the predictability of eruption pathways, SME may help reduce the risk of adjacent root resorption and root dilaceration. Notably, its effects are more pronounced once the ectopic canine has progressed beyond the lateral aspect of the lateral incisor roots, suggesting that this positional reference may serve as a critical reference point for determining the optimal timing of SME intervention.

## Figures and Tables

**Table 1:** The distribution of gender, age, and follow-up period among the treatment and control groups.

		Treatment Group	Control Group	*p* Value
Sex	Male	29	22	0.56 *
	Female	47	36	
Age at T0 (years)	Mean ± SD	8.38 ± 0.89	8.46 ± 0.79	0.63 ^#^
Follow-up period (months)	Mean ± SD	11.04 ± 4.44	10.32 ± 3.96	0.33 ^#^

* Chi-square test, ^#^ Mann–Whitney U test and Independent Samples *t*-test were applied for inter-group comparisons.

**Table 2:** The distribution of sectors before and after treatment/observation among the treatment and control groups.

	Treatment Group		Control Group		*p* Value (Treatment Group vs. Control Group)
	sec.1	sec.234	sec.1	sec.234	
T0	112	40	84	32	0.816
T1	131	21	100	16	0.996
T1 − T0	19	−19	16	−16	0.833
*p* value (T0 vs. T1)	0.007		0.01		

Statistical analysis: The chi-square test was applied for intra-group comparisons. T0: Baseline (pre-treatment/observation period); T1: Post-intervention/follow-up assessment.

**Table 3:** Intragroup comparisons of the canine position at T0 and T1, as well as the mean change (T1 − T0).

Group	Time Point	3c-OP (Proportion)		3c-ML (Proportion)		3^ML (°)	
		Mean	SD	Mean	SD	Mean	SD
Treatment Group (*n =* 152)	T0	18.63	5.86	18.91	2.78	11.27	8.38
	T1	13.68	7.04	20.38	3.55	7.06	10.67
	Change T1 − T0	−4.951	4.854	1.468	3.117	−4.209	8.187
	*p* value	0.000		0.000		0.000	
Control Group (*n =* 116)	T0	18.54	5.07	19.84	3.05	10.59	9.46
	T1	14.46	6.21	20.84	3.35	8.80	10.00
	Change T1 − T0	−4.081	4.085	0.968	2.455	−1.789	7.820
	*p* value	0.000		0.000		0.015	
TE (*n =* 40)	T0	19.47	4.55	16.40	2.53	16.21	8.68
	T1	16.71	5.29	18.46	3.78	12.01	12.83
	Change T1 − T0	−2.76	4.56	2.06	3.26	−4.21	9.10
	*p* value	0.000		0.000		0.006	
CE (*n =* 32)	T0	20.71	3.75	17.89	3.13	16.76	10.88
	T1	16.44	4.70	18.49	3.40	15.36	10.30
	Change T1 − T0	−4.27	4.08	0.50	2.01	−1.40	7.91
	*p* value	0.000		0.095		0.324	
TN (*n =* 112)	T0	18.34	6.25	19.75	2.38	9.50	7.56
	T1	12.60	7.29	21.13	3.09	5.29	9.23
	Change T1 − T0	−5.74	4.73	1.38	2.98	−4.21	7.88
	*p* value	0.000		0.000		0.000	
CN (*n =* 84)	T0	17.71	5.27	20.58	2.69	8.24	7.71
	T1	13.70	6.57	21.74	2.88	6.30	8.72
	Change T1 − T0	−4.01	4.11	1.15	2.59	−1.94	7.83
	*p* value	0.000		0.000		0.356	
TES (*n =* 15)	T0	20.40	4.73	18.06	2.27	13.04	10.70
	T1	18.31	4.18	18.44	2.49	12.13	11.21
	Change T1 − T0	−2.09	4.94	0.38	1.94	−0.91	7.05
	*p* value	0.124		0.456		0.761	
TEI (*n =* 25)	T0	18.90	4.45	15.41	2.23	12.38	10.59
	T1	15.75	5.71	18.48	4.49	12.34	10.00
	Change T1 − T0	−3.16	4.37	3.06	3.57	−0.04	8.25
	*p* value	0.001		0.000		0.003	
CES (*n =* 24)	T0	21.12	4.06	18.07	3.13	10.34	6.75
	T1	16.77	5.07	18.56	3.15	4.42	9.40
	Change T1 − T0	−4.36	4.16	0.49	1.76	−5.92	8.24
	*p* value	0.000		0.186		0.774	
CEI (*n =* 8)	T0	19.47	2.40	17.34	3.26	9.13	8.23
	T1	15.47	3.49	18.27	4.31	5.92	9.11
	Change T1 − T0	−4.00	4.10	0.93	2.63	−3.21	7.20
	*p* value	0.028		0.350		0.199	

Statistical analysis: The paired-samples *t*-test and Wilcoxon signed-rank test were applied for intra-group comparisons, while the independent samples *t*-test was used for intergroup comparisons. TE refers to ectopic canines in the treatment group; TN indicates normally positioned canines in the treatment group. CE denotes ectopic canines in the control group, and CN represents normally positioned canines in the control group. Subgroup classifications include TES (superior-positioned canines within TE), TEI (inferior-positioned canines within TE), CES (superior-positioned canines within CE), and CEI (inferior-positioned canines within CE).

**Table 4:** Intergroup comparisons of the change values (T1 − T0).

Group	3c-OP (Proportion) (T1 − T0)		3c-ML (Proportion) (T1 − T0)		3^ML° (T1 − T0)	
	Mean Difference (95%CI)	*p* Value	Mean Difference (95% CI)	*p* Value	Mean Difference (95% CI)	*p* Value
TE vs. CE	1.513 (−0.55, 3.57)	0.147	1.56 (0.29, 2.82)	0.043	−2.81 (−6.87, 1.26)	0.173
TES vs. CES	−2.27 (−5.25, 0.71)	0.131	−0.11 (−1.33, 1.12)	0.861	−0.07 (−4.92, 4.62)	0.976
TES vs. TEI	−1.07 (−4.10, 1.96)	0.478	−2.68 (−4.71, −0.65)	0.011	5.88 (0.11, 11.66)	0.046

Mann–Whitney U test and Independent Samples *t*-test were applied for inter-group comparisons. TE refers to ectopic canines in the treatment group; CE denotes ectopic canines in the control group, TES (superior-positioned canines within TE), TEI (inferior-positioned canines within TE), CES (superior-positioned canines within CE).

## Data Availability

Data supporting the reported outcomes are available from the corresponding author upon reasonable request. The data are not publicly available due to ethical restrictions related to participant confidentiality and privacy protections.

## Abbreviations

The following abbreviations are used in this manuscript:

SME	Slow maxillary expansion
RME	Rapid maxillary expansion
OPGs	Panoramic radiographs
3c-OP	Canine cusp-to-occlusal plane distance
3c-ML	Canine cusp-to-midline distance
3^ML	Canine-to-midline angle
TE	Ectopic canine in treatment group
TN	Normally positioned canine in treatment group
CE	Ectopic canine in control group
CN	Normally positioned canine in control group
TES	Superior-positioned canines in the TE group
TEI	Inferior-positioned canines in the TE group
CES	Superior-positioned canines in the CE group
CEI	Inferior-positioned canines in the CE group
ICC	Intraclass correlation coefficients
